# The Protective Effect of Basic Fibroblast Growth Factor on Diabetic Nephropathy Through Remodeling Metabolic Phenotype and Suppressing Oxidative Stress in Mice

**DOI:** 10.3389/fphar.2020.00066

**Published:** 2020-02-21

**Authors:** Tingting Wei, Qi Shu, Jie Ning, Shuaijie Wang, Chen Li, Liangcai Zhao, Hong Zheng, Hongchang Gao

**Affiliations:** ^1^ School of Pharmaceutical Sciences, Wenzhou Medical University, Wenzhou, China; ^2^ Laboratory Animal Centre, Wenzhou Medical University, Wenzhou, China

**Keywords:** bFGF, diabetes, metabolomics, nephropathy, taurine, oxidative stress

## Abstract

Diabetic nephropathy is a common complication in diabetes, but still lack of effective therapeutic strategies. This study aimed to investigate the therapeutic effect of basic fibroblast growth factor (bFGF) in *db/db* mice with diabetic nephropathy and explore its possible metabolic mechanisms using a nuclear magnetic resonance-based metabolomic approach. We found that bFGF treatment significantly alleviate urinary albumin to creatinine ratio and renal fibrosis in *db/db* mice, suggesting a potential renal protective effect. Metabolomics results reveal that bFGF remodeled metabolic phenotypes of the kidney and urine in *db/db* mice, mainly involving energy metabolism, methylamine metabolism, osmoregulation, and oxidative stress. Furthermore, the results show that bFGF-induced reductions of oxidative stress and apoptosis in *db/db* mice might be mediated by NOX-ROS-Nrf2 signaling. Therefore, our study suggests that the protective effect of bFGF on diabetic nephropathy could be mediated by remodeling metabolic phenotype and suppressing oxidative stress.

## Introduction

Diabetic nephropathy (DN) is one of the most common microvascular complications of diabetes and has become the leading cause of end-stage renal failure and death of both type I and II diabetic patients (Yu and Bonventre, 2018; Wang et al., 2019). The progression of DN involve several stages, such as glomerular hypertrophy, proteinuria, glomerulosclerosis, interstitial fibrosis, and renal failure (Li et al., 2019). Several potential mechanisms underlying DN development have been reported, including oxidative stress (Barman et al., 2018), inflammation (Olatunji et al., 2018), advanced glycation end products, and polyol pathway (Pan et al., 2010). However, there is still a lack of effective treatments for DN, so elucidating the mechanisms and discovering new drugs of DN treatment are urgently required.

Basic fibroblast growth factor (bFGF), a member of the growth factor family, has been reported to reduce the functional and morphological damages in chronic kidney disease and induce the re-expression of nephrogenic/angiogenic factors (Villanueva et al., 2014). bFGF treatment can effectively reduce serum glucose and lipid levels in STZ-induced diabetic rats and prevent DN through inhibition of inflammation (Lin et al., 2016; Sheng et al., 2018). Additionally, Tan *et al*. reported that bFGF protects against renal ischemia reperfusion injury by attenuating mitochondrial damage and proinflammatory signaling (Tan et al., 2017). Of note, a recent review revealed that the FGF family possesses multifarious roles in metabolic homeostasis (Li, 2019). Yet, the metabolic mechanisms of bFGF on diabetic kidney diseases remain unclear.

Metabolomics is an omics approach that aims to analyze a comprehensive set of low-molecular weight metabolites in biological samples under pathophysiological conditions (Nagana Gowda and Raftery, 2017). It has been extensively applied for identifying potential biomarkers and exploring pathogenesis of diseases (Nicholson et al., 1999; Lu et al., 2013; Chen et al., 2014). Nuclear magnetic resonance (NMR) spectroscopy is a common analytical technique used in metabolomics studies owing to its advantages, such as simple sample preparation, rapid analysis, and high reproducibility. In our previous studies, we have reported several defective metabolic pathways in the kidney of diabetic mice and rats, such as TCA cycle, glycolysis, methylamine pathway, fatty acids β-oxidation, ketogenesis, and glycogenic amino acid pathway (Guan et al., 2013; Wei et al., 2015). Moreover, Zhao *et al.* have found that abnormal energy metabolism in the kidney of rats was associated with the pathogenic process of DN (Zhao et al., 2011).

In the present study, we analyzed metabolic profiles of the kidney and urine in type 2 diabetic *db/db* mice with DN after bFGF treatment by using a ^1^H NMR-based metabolomic approach. The purposes of this study are (1) to examine the therapeutic effect of bFGF on DN, and (2) to explore its potential metabolic mechanisms.

## Materials and Methods

### Animals

Eight-week-old male *db/db* (C57BLKS/J-lepr^db^/lepr^db^) mice and age-matched wild-type (wt) mice were purchased from the Model Animal Research Center of Nanjing University (Nanjing, China). Mice were housed in specific pathogen-free (SPF) colony under a fully controlled condition (room temperature, 22 ± 2°C; humidity, 50–60%; light/dark, 12 h/12 h) at the Laboratory Animal Center of Wenzhou Medical University (WMU, Wenzhou, China). All mice were given free access to standard rat chow and tap water. The present study was conducted on the basis of the Guide for the Care and Use of Laboratory Animals and approved by the Institutional Animal Care and Use Committee of WMU.

### bFGF Treatment

**Figure 1:**
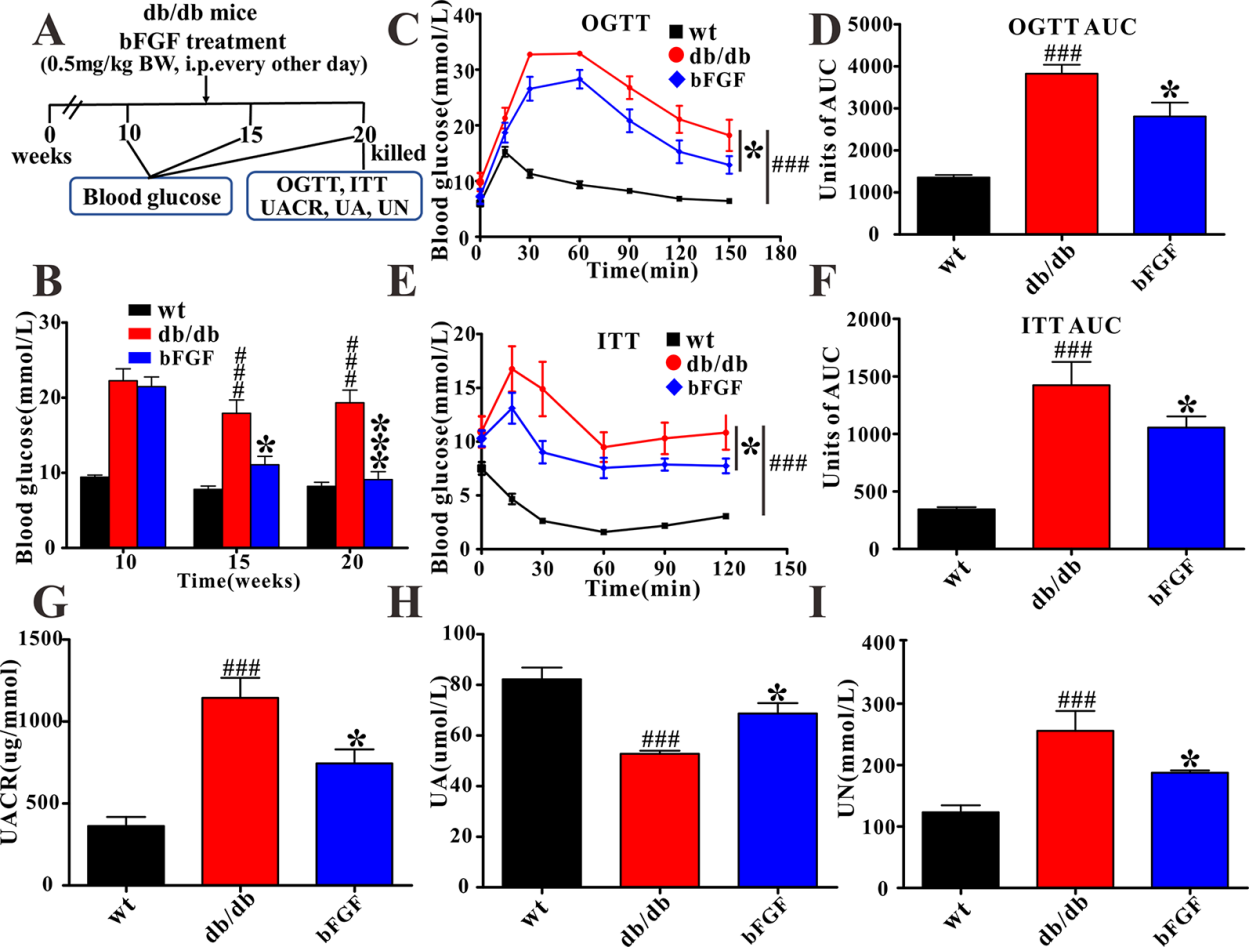
The protective effect of bFGF on diabetic nephropathy in *db/db* mice. **(A)** bFGF treatment procedure for *db/db* mice. Arrows indicate when mice received i.p. injection of vehicle or bFGF (0.5 mg/kg BW) for10 weeks and then were killed at 20 weeks for study. **(B, C)** Oral glucose tolerance test in wild-type (wt), *db/db*, and bFGF-treated mice. **(D)** Random blood glucose level of wt, *db/db*, and bFGF-treated mice. **(E, F)** Insulin tolerance test in wt, *db/db*, and bFGF-treated mice. **(G, H, I)** The levels of urinary albumin to creatinine ratio, uric acid, and urea nitrogen in urine of wt, *db/db*, and bFGF-treated mice. Significant level: ^###^ *P* < 0.001 versus wildtype (wt) mice; *P < 0.05 and ***P < 0.001 versus *db/db* mice.

All mice were acclimatized for 1 week and then randomly divided into bFGF-treated and control groups at 10 weeks of age. For bFGF group, *db/db* mice were intraperitoneally (i.p.) injected with bFGF at a dose of 0.5 mg/kg body weight every other day for 10 weeks (**Figure 1A**). The dose of bFGF treatment was selected according to the previous publication (Tan et al., 2017). Meanwhile, the wt mice and *db/db* mice in the control group received 0.9% saline using the same schedule.

### Urine and Kidney Sample Collection

Urine samples were individually collected from mice before sacrifice in metabolic cages for 12 h. The urine samples were added with 0.1 ml of 1% sodium azide solution for avoiding bacterial contamination and then centrifuged at 3,000*g* at 4°C for 10 min. The supernatant was transferred into a new tube and stored at -80°C until analysis. Mice were sacrificed by cervical dislocation at 20 weeks of age, and their renal tissues were isolated, immediately snap-frozen in liquid nitrogen, and kept at -80°C until use.

### Biochemical Analysis and Histopathological Examination

In this study, blood glucose level was measured using the Precision G Blood Glucose Testing System (Abbott Laboratories, Abbott Park, IL). Oral glucose tolerance test (OGTT) and insulin tolerance test (ITT) were performed on mice after 10 weeks of bFGF treatment (**Figure 1A**). After a 12 h fast, mice were orally administered with 50% glucose solution at a dose of 2 g/kg body weight. For ITT, mice were i.p. injected with insulin solution at a dose of 0.35 U/kg body weight after a 4 h fast. Blood glucose levels were measured from the tail vein at time 0, 30, 60, 90, 120, and 150 min. The levels of urea nitrogen (UN), uric acid (UA), urinary albumin to creatinine ratio (UACR) in the urine was determined using an automatic biochemistry analyzer (Mindray BS-300).

For pathologic examination, the kidney tissues were harvested, fixed overnight in 4% paraformaldehyde, and then embedded in paraffin. After deparaffinization and rehydration, the paraffin sections (5 mm) were stained with hematoxylin and eosin (HE) for routine histopathological observations. Periodic acid-Schiff (PAS) and Masson’s trichrome staining were used to determine collagen deposition and fibrosis, respectively.

### RNA Extraction, cDNA Synthesis, and Quantitative RT-PCR

Total RNA was extracted from renal tissues with Trizol reagent (Invitrogen, Carlsbad, CA). The purity of RNA was measured by a nanodrop spectrometry (Thermo Fisher Scientific, Beverly, MA) and expressed as the ratio of OD values at 260/280 nm. An OD 260/280 ratio greater than 1.80 represents high RNA purity. Then, RNA was used to synthesize the first-strand cDNAs by the Prime Script™ RT Reagent Kit (TaKaRa, Kusatsu, Japan). The quantification process was carried out in a 10 μl final reaction volume using a SYBR Green PCR Master Mix (Bio-Rad, CA, USA). In this study, GAPDH served as an endogenous control, and the primers were synthesized by Sunny Biotechnology (Sunny, Shanghai, China).

### Western Blot Analysis

The renal tissues (30–40 mg) were lysed with RIPA buffer (25 mM Tris, pH 7.6, 150 mM NaCl, 1% NP-40, 1% sodium deoxycholate, and 0.1% SDS) supplemented with protease and phosphatase inhibitors (Thermo Fisher Scientific, MA). Total protein concentration was measured using the BCA protein assay kit (Bio-Rad, CA, USA). After normalization, equal amounts of proteins were separated by 10% SDS–PAGE and transferred to PVDF membranes (0.45 μm, Millipore, Germany). The membranes were blocked with 5% nonfat milk in TBST for 2 h and incubated overnight with primary antibodies at 4°C. After three washes with TBST, the membranes were incubated with secondary antibodies (Thermo Fisher Scientific, 1:10,000) at room temperature for 1 h. Finally, the blots were washed with TBST three times and incubated using the EasySee western Blot Kit (Transgen Biotech, China) to visualize the immunoreactive bands.

### Sample Preparation and NMR Measurement

^1^H NMR spectra were acquired at 25°C on a Bruker AVANCE III 600 MHz NMR spectrometer equipped with a triple resonance probe and a z-axis pulsed field gradient (Bruker BioSpin, Rheinstetten, Germany). Prior to NMR analysis, urine samples were thawed, and 200 μl aliquots of the samples were mixed with 50 μl D_2_O containing sodium trimethylsilyl propionate-d_4_ (TSP, 0.36 mg/ml) and 300 μl of phosphate buffer (0.2 M Na_2_HPO_4_/NaH_2_PO_4_, pH 7.4) to minimize pH variations (Xiao et al., 2009). The mixtures were centrifuged and then 500 μl of the supernatant was transferred to 5-mm NMR tubes for metabolomics analysis. One-dimensional NOESY pulse sequence with water signal pre-saturation was performed to acquire NMR spectra of urine samples. The main acquisition parameters were set as follows: spectral width = 12,000 Hz; data points = 256 K; relaxation delay = 4 s; acquisition time = 2.66 s per scan.

The frozen renal tissues were weighed into a centrifuge tube. Then, ice-cold methanol (4 ml/g) and distilled water (0.85 ml/g) was added into the tube, homogenized at 4°C after thawing and mixed by vortex for 15 s. Subsequently, ice-cold chloroform (2 ml/g) and distilled water (2 ml/g) was added into the tube and mixed again for 15 s. The mixture were kept on ice for 15 min and centrifuged at 10,000*g* for 15 min at 4°C. The supernatant was extracted into a new tube and lyophilized for about 24 h. The lyophilized extract was reconstituted in 500 μl D_2_O containing TSP and transferred to 5 mm NMR tubes for analysis. A one-dimensional ZGPR pulse sequence with water signal presaturation was used to acquire NMR data. In addition, the main acquisition parameters were set as follows: data points = 256 K; spectral width = 12,000 Hz; relaxation delay = 4 s; acquisition time = 2.66 s per scan.

### Multivariate Data Analysis and Statistical Analysis

All NMR spectra were manually corrected for phase/baseline and referenced to TSP peak at 0 ppm using TopSpin 3.0 software (Bruker BioSpin, Rheinstetten, Germany). Then, the “icoshift” procedure was employed to align NMR spectra under the MATLAB environment (R2012a, The MathWorks Inc., Natick, MA, USA) (Savorani et al., 2010). The spectral region from 0.0 to 9.0 ppm excluding the residual water signals (4.65–5.05 ppm for kidney extract; 4.78–4.83 ppm for urine) were subdivided and integrated to binning data with a size of 0.01 ppm for further multivariate analysis.

To discriminate metabolic patterns between different groups, partial least squares-discriminant analysis (PLS-DA) was performed using Pareto-scaled NMR data in SIMCA 12.0 software (Umetrics, Umeå, Sweden). Leave one-out cross validation and permutation tests (200 cycles) were used to examine the performance of the model. PLS-DA loading plots were used to identify the important metabolites for the separation of groups. The significance of metabolites in PLS-DA was assessed using the absolute value of the correlation coefficient, |*r*|, and high |*r*| value was considered important.

The differences in metabolite levels between two groups were analyzed using independent-samples *t*-tests with SPSS software (version 13.0; SPSS Inc., Chicago, IL, USA). Additionally, the differences in OGTT and ITT between two groups were assessed with repeated measure ANOVA in SPSS 13.0 software. A statistically significant difference was considered when *P* value <0.05.

## Results

### The Protective Effect of bFGF on Diabetic Nephropathy in *db/db* Mice

In this study, as expected, *db/db* mice at 10 weeks of age exhibited a higher level of blood glucose than age-matched wild-type (wt) mice (**Figure 1B**). Of note, we found that blood glucose level was significantly reduced in *db/db* mice after 5 and 10 weeks of bFGF treatment, as shown in **Figure 1B**. Furthermore, OGTT demonstrated that administration of bFGF significantly improved blood glucose clearance in *db/db* mice (**Figures 1C, D**). As can be seen from insulin tolerance test, insulin sensitivity was also enhanced in *db/db* mice with bFGF treatment (**Figures 1E, F**). To evaluate renal function of mice, we measured UACR, which is a clinical marker of kidney damage and dysfunction (Teimoury et al., 2014). The result shows that the UACR level was significantly increased in *db/db* mice compared with age-matched wt mice (**Figure 1G**), whereas bFGF treatment significantly decreased its level in *db/db* mice. Additionally, uric acid and urea nitrogen are another two hallmarks of renal injury (Barutta et al., 2010). We found that the UA level was significantly reduced in *db/db* mice relative to age-matched wt mice (**Figure 1H**). However, interestingly, its level was significantly increased after bFGF treatment and reached to the normal level. In addition, *db/db* mice had a higher UN level than age-matched wt mice, but this increase can be recovered after bFGF treatment (**Figure 1I**).

**Figure 2:**
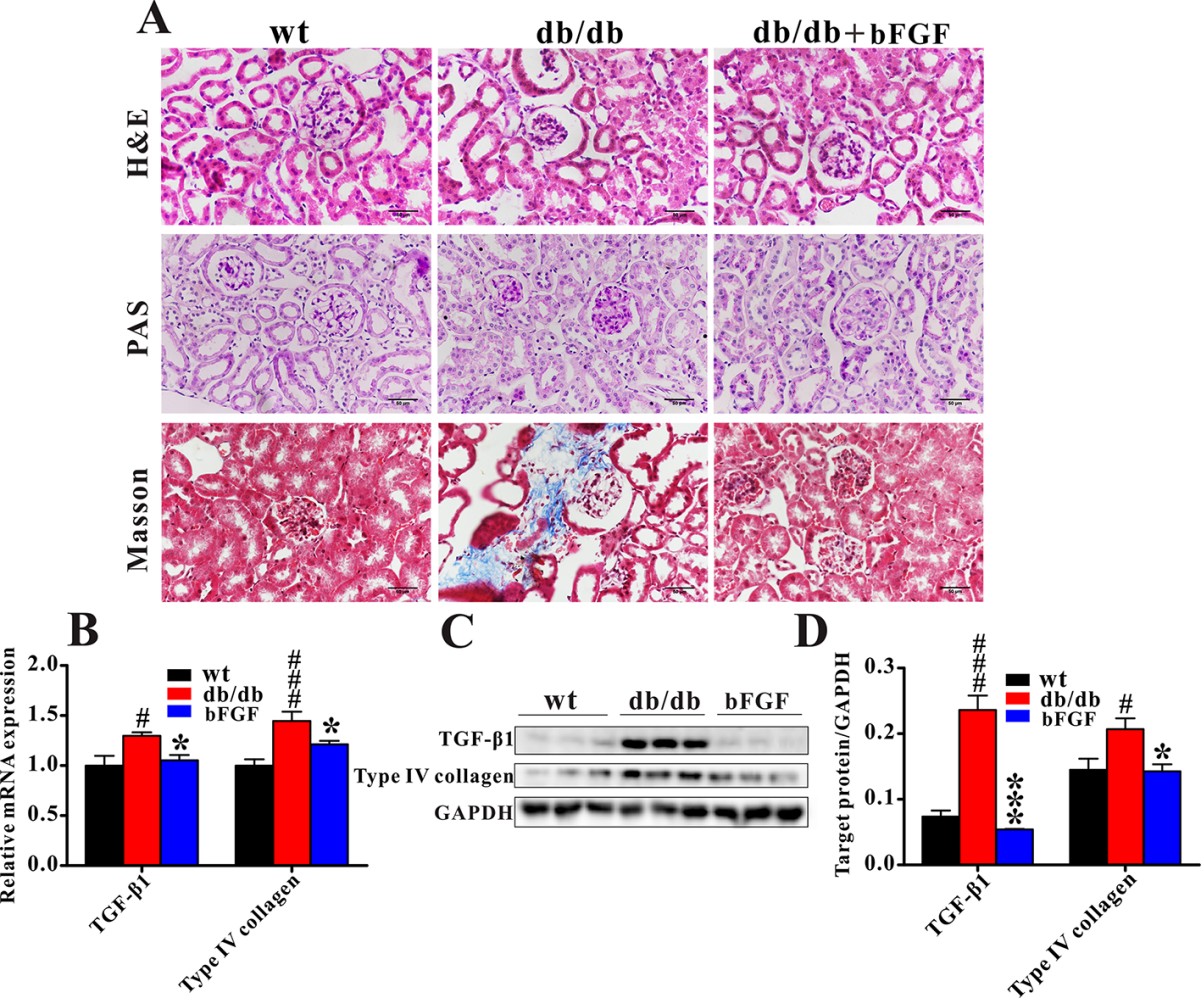
bFGF recovered renal fibrosis in *db/db* mice. **(A)** Representative images of renal tissues stained with H&E and PAS (indicating glycogen) for evaluation of mesangial expansion, and Masson trichrome for type IV collagen. Bar = 50 μm. **(B)** The relative mRNA expression of TGF-β1 and type IV collagen in renal tissues of wild-type (wt), *db/db*, and bFGF-treated mice. **(C)** Western blot analysis of TGF-β1 and type IV collagen in renal tissues of wt, *db/db*, and bFGF-treated mice. **(C)** Intensities of TGF-β1 and type IV collagen normalized to GADPH. Significant level: ^#^ *P* < 0.05 and ^###^ *P* < 0.001 versus wt mice; *P < 0.05 and ***P < 0.001 versus *db/db* mice.

**Figure 2A** shows that *db/db* mice displayed notable glomerular hypertrophy and mesangial matrix expansion as compared with age-matched wt mice, while these symptoms were markedly alleviated after bFGF treatment. In addition, Masson trichrome staining revealed a significant increase in renal fibrosis in *db/db* mice, but this change cannot be observed in *db/db* mice treated with bFGF. Consistent with our histological findings, the relative mRNA expression levels of profibrotic molecules transforming growth factor (TGF) β1 and type IV collagen were significantly increased in the kidney of *db/db* mice relative to age-matched wt mice; however, of note, these two indicators were recovered to the normal level after bFGF treatment (**Figure 2B**). Moreover, bFGF treatment induced parallel changes in the protein expression of these two profibrotic markers in renal tissues (**Figures 2C, D**). Taken together, these findings revealed that bFGF had a potential protective effect on diabetic nephropathy in *db/db* mice.

### Changes of Metabolic Phenotypes in the Kidney and Urine in *db/db* Mice Treated With bFGF

**Figure 3:**
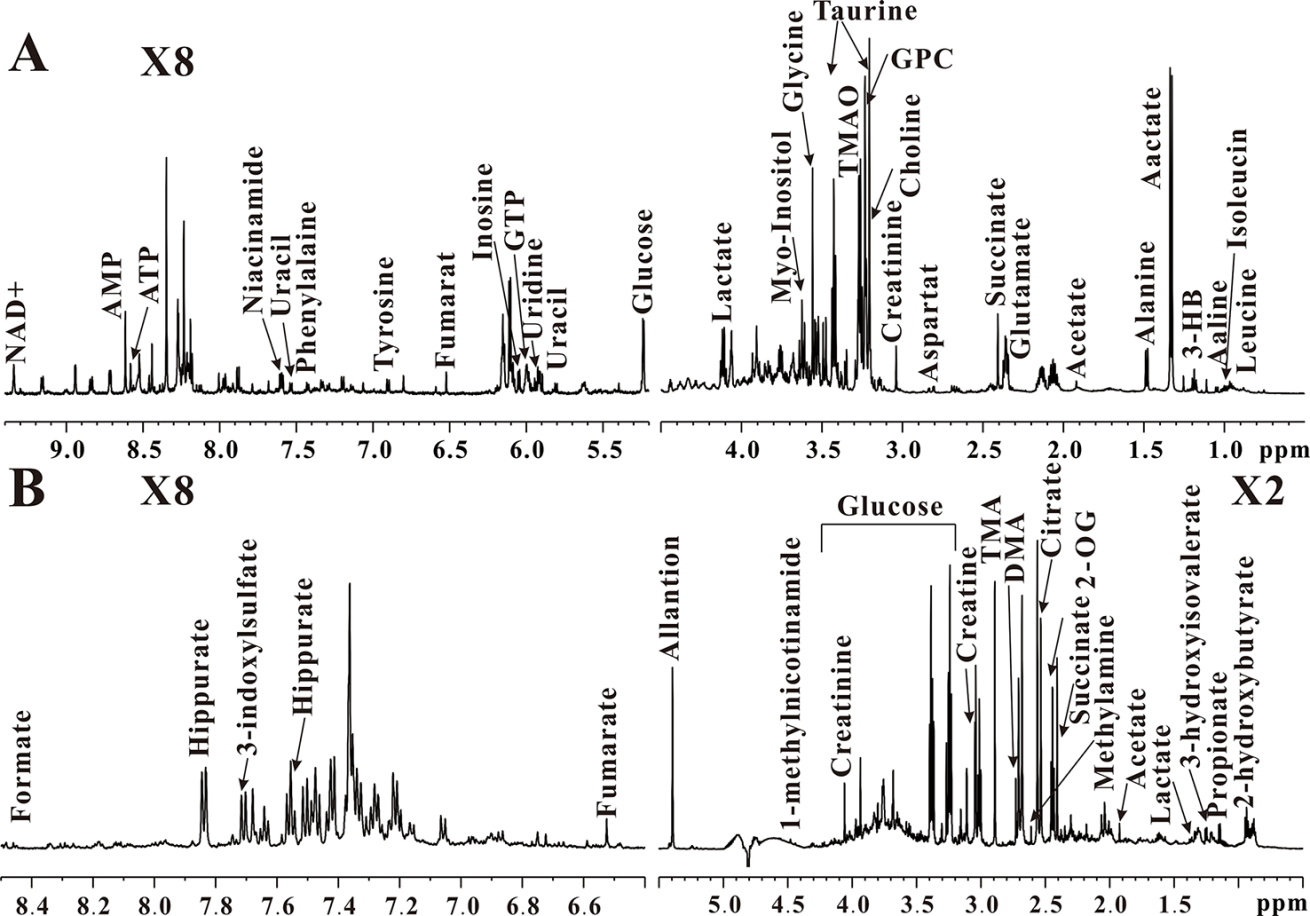
Typical 600 MHz ^1^H NMR spectra of kidney **(A)** and urine **(B)** in wild-type mice.

Typical ^1^H NMR spectra obtained from the kidney and urine samples of wt mice are illustrated in **Figures 3A, B**, respectively. In total, 43 metabolites were identified from NMR-based metabolome, mainly including energy metabolism-related metabolites (succinate, 2-oxoglutarate, citrate, creatine, creatinine, lactate, glucose, fumarate, ATP, AMP, and NAD+), amino acids (leucine, isoleucine, valine, alanine, glutamate, aspartate, taurine, glycine, tyrosine, and phenylalanine), methylamine metabolites (methylamine, dimethylamine, trimethylamine, choline, and TMAO), ketobodies (3-hydroxyisovalerate, 3-hydroxybutyrate, and 2-hydroxybutyrate), nucleotide metabolism-related metabolites (uracil, uridine, GTP, and allantion), short-chain fatty acids (formate, acetate, and propionate), and others (GPC, myo-inositol, inosine, niacinamide, 1-methylnicotinamide, hippurate, and 3-indoxylsulfate).

**Figure 4:**
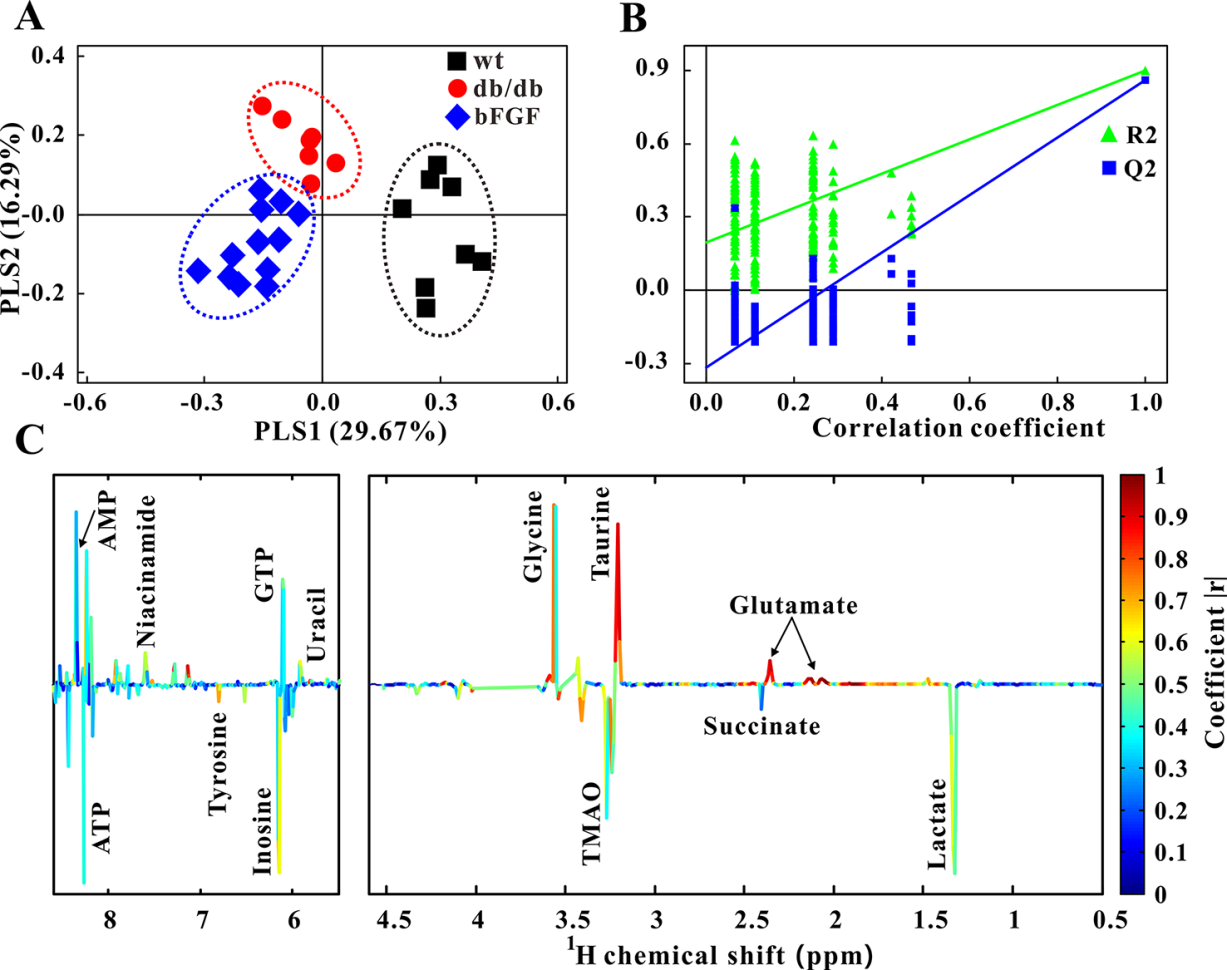
Changes of metabolic phenotypes in kidney of *db/db* mice treated with bFGF. **(A)** PLS-DA score plot (*R* ^2^X = 0.46, *R* ^2^Y = 0.747, *Q* ^2^ = 0.624, *P* < 0.001). **(B)** Permutation test (200 cycles, *R* ^2^ = 0.897, *Q* ^2^ = 0.858). **(C)** Coefficient-coded loading plot.

**Figure 5:**
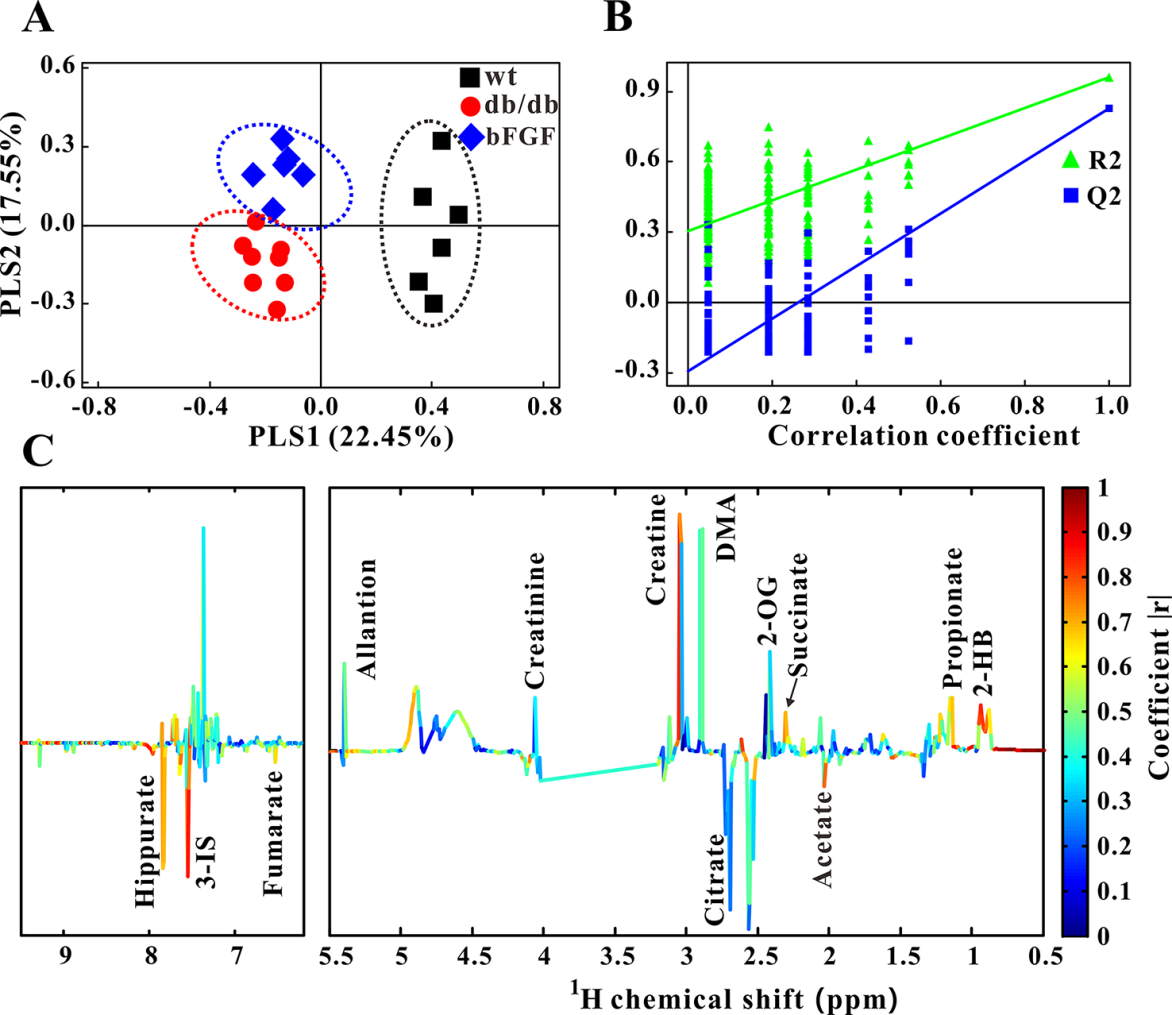
Changes of metabolic phenotypes in urine of *db/db* mice treated with bFGF. **(A)** PLS-DA score plot (*R* ^2^X = 0.4, *R* ^2^Y = 0.745, *Q* ^2^ = 0.491, *P* < 0.001). **(B)** Permutation test (200 cycles, *R* ^2^ = 0.961, *Q* ^2^ = 0.828). **(C)** Coefficient-coded loading plot.

Furthermore, PLS-DA was used to examine changes in metabolic patterns among wt, *db/db*, and bFGF groups and identify key metabolites. The PLS-DA score plot showed a clear separation among these three groups (**Figure 4A**), which is validated by a permutation test (**Figure 4B**). According to its loading plot, a series of metabolites that contribute to this separation were identified, such as lactate, succinate, ATP, AMP, glutamate, taurine, glycine, tyrosine, uracil, GTP, TMAO, inosine, and niacinamide, as shown in **Figure 4C**. However, of note, taurine showed the most significant contribution. In addition, PLS-DA score plot based on urine metabolome also showed a clear difference among these three groups (**Figure 5A**) which is validated by a permutation test (**Figure 5B**). The corresponding loading plot identified a series of important metabolites, including acetate, succinate, 2-oxoglutarate, citrate, creatine, creatinine, fumarate, hydroxybutyrate, DMA, propionate, allantion, hippurate, and 3-indoxylsulfate (**Figure 5C**).

### Metabolic Changes in the Kidney and Urine of *db/db* Mice Treated With bFGF

**Figure 6:**
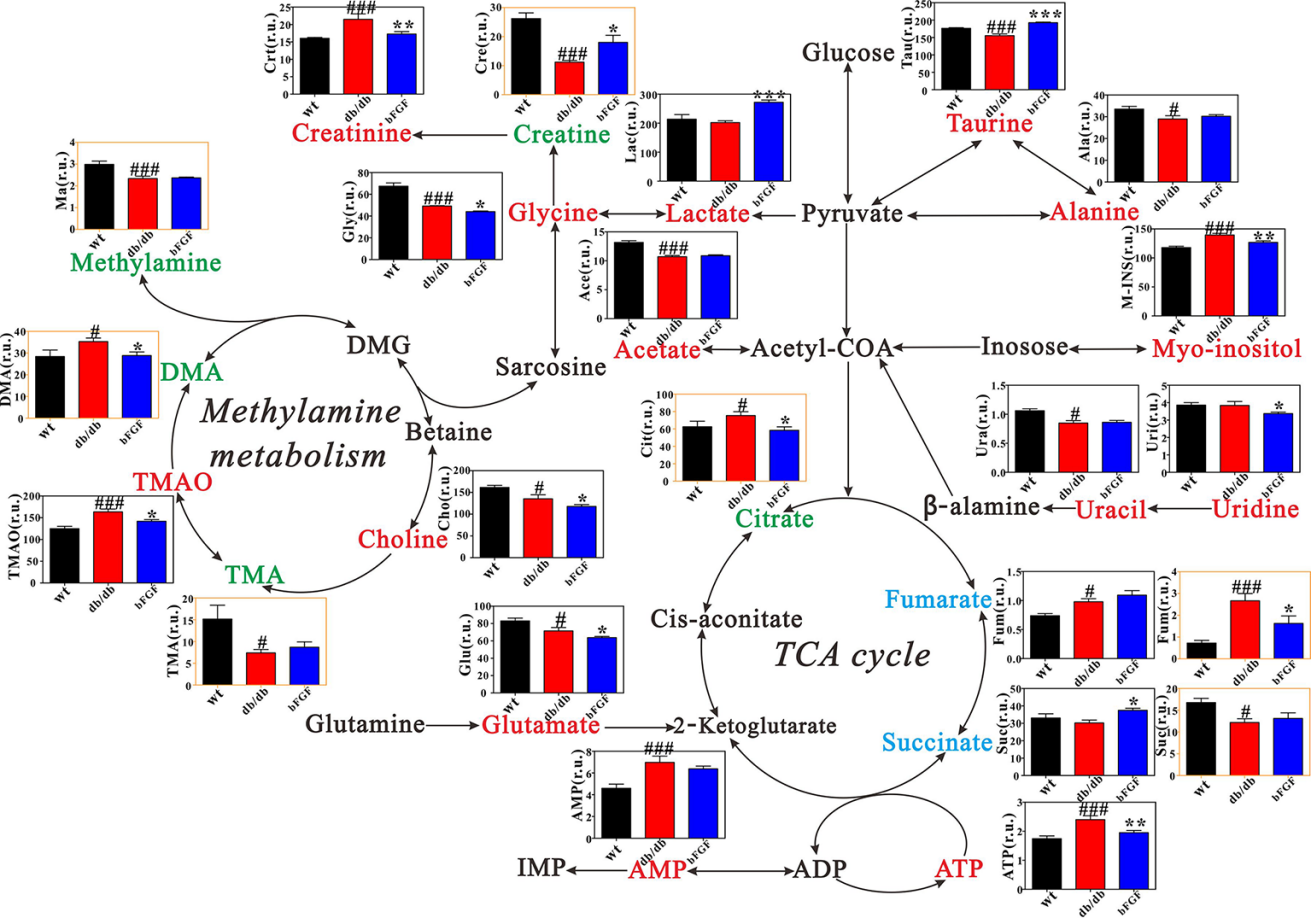
Metabolic changes in kidney and urine of *db/db* mice treated with bFGF. Red and green texts indicate metabolites detected from kidney and urine samples, respectively. Blue text indicates metabolites detected from both kidney (black outline box) and urine (yellow outline box) samples. Metabolites: Tau, taurine; Ala, alanine; Crt, creatinine; Cre, creatine; Lac, lactate; Ace, acetate; M-INS, myo-inositol; Ura, uracil; Uri, uridine; Gly, glycine; Ma, methylamine; DMA, dimethylamine; TMA, trimethylamine; TMAO, Trimethylamine oxide; Cho, choline; Cit, citrate; Fum, fumarate; Suc, succinate; Glu, glutamate. r.u., relative unit. Significant level: ^#^ *P* < 0.05 and ^###^ *P* < 0.001 versus wt mice; *P < 0.05, **P < 0.01, and ***P < 0.001 versus *db/db* mice.

**Figure 6** shows the metabolic pathway changes in *db/db* mice after bFGF treatment. We found that the levels of creatinine, citrate, ATP, fumarate, TMAO, DMA, and myo-inositol were significantly increased in *db/db* mice compared with age-matched wt mice, but these increased trends were significantly reduced after bFGF treatment (**Figure 6**). Moreover, *db/db* mice had significantly lower creatine and taurine levels than age-matched wt mice, while bFGF treatment significantly increased their levels, as shown in **Figure 6**. Relative to age-matched wt mice, we also found that *db/db* mice had significantly decreased levels of alanine, uracil, acetate, glycine, methylamine, TMA, choline, and glutamate as well as increased levels of fumarate and AMP in the kidney, but no significant alterations were observed after bFGF treatment. Together, these metabolites mainly involved energy metabolism, amino acid metabolism, and methylamine metabolism.

### bFGF Suppress Oxidative Stress in the Kidney of *db/db* Mice

**Figure 7:**
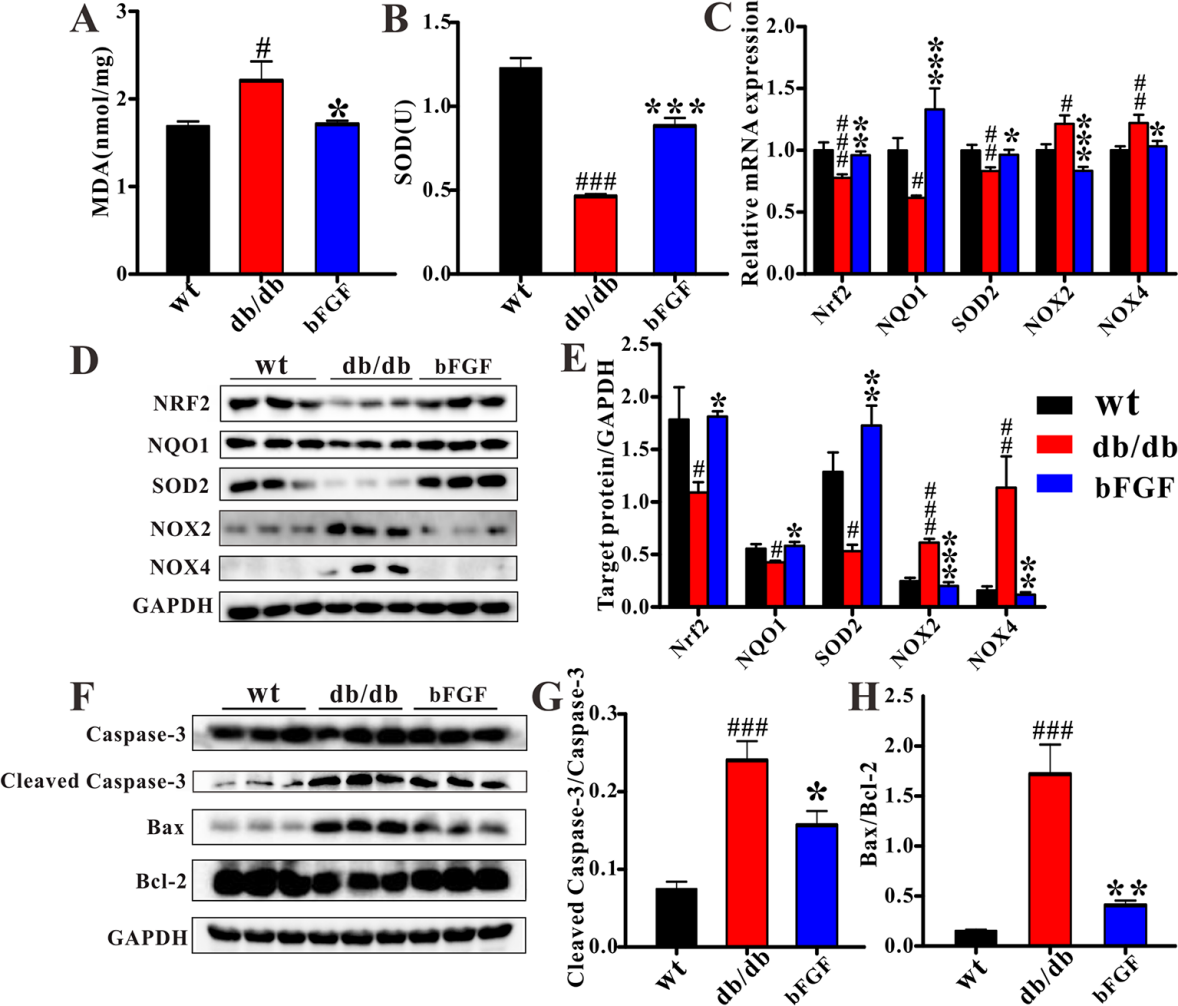
bFGF suppressed oxidative stress and apoptosis in kidney of *db/db* mice. The levels of **(A)** MDA and **(B)** SOD in renal tissues of wild-type (wt), *db/db*, and bFGF-treated mice. **(C)** The relative mRNA expression of Nrf2, NQO1, SOD2, NOX2, and NOX4 in renal tissues of wt, *db/db*, and bFGF-treated mice. **(D)** Western blot analysis of Nrf2, NQO1, SOD2, NOX2, and NOX4 in renal tissues of wt, *db/db*, and bFGF-treated mice. **(E)** Intensities of Nrf2, NQO1, SOD2, NOX2, and NOX4 normalized to GADPH. **(F)** Western blot analysis of caspase-3, cleaved caspase-3, Bax, and Bcl-2 in renal tissues of wt, *db/db*, and bFGF-treated mice. **(G)** Intensities of cleaved caspase-3 normalized to caspase-3. **(H)** Intensities of Bax normalized to Bcl-2. Significant level: ^#^ *P* < 0.05, ^##^ *P* < 0.01, and ^###^ *P* < 0.001 versus wt mice; *P < 0.05, **P < 0.01, and ***P < 0.001 versus *db/db* mice.

**Figure 8:**
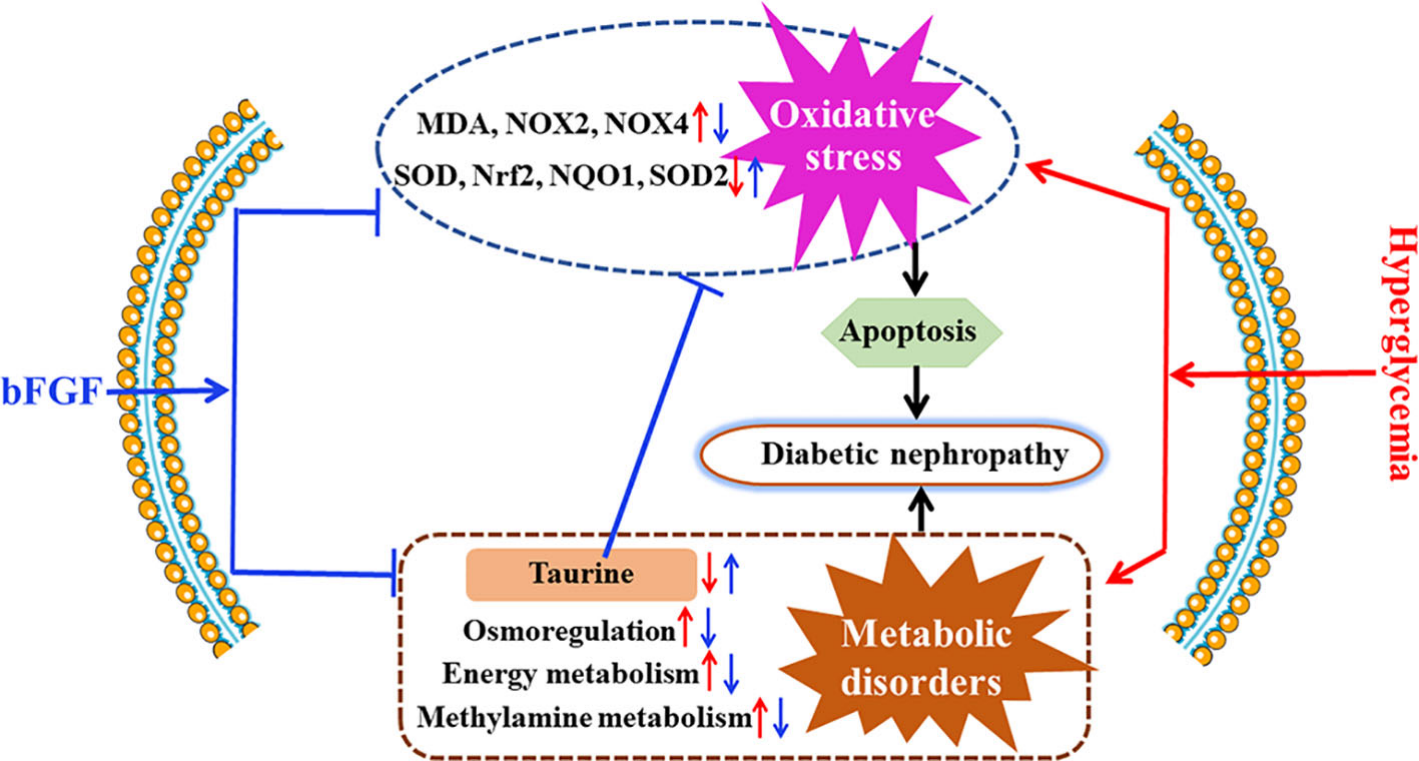
Potential mechanisms for the protective effect of bFGF on diabetic nephropathy. bFGF treatment corrected metabolic disorders and suppressed oxidative stress in *db/db* mice with diabetic nephropathy. Red arrow indicates changes in diabetic nephropathy and blue arrow indicates changes after bFGF treatment.

To investigate whether bFGF can suppress oxidative stress, several relevant markers were detected in the kidney of *db/db* mice, as shown in **Figure 7**. As compared with age-matched wt mice, *db/db* mice had a significantly increased MDA level (**Figure 7A**) and a significantly decreased SOD level (**Figure 7B**). However, of note, these two markers were recovered to the normal level after bFGF treatment. In addition, the relative mRNA expression levels of antioxidant biomarkers, including nuclear factor erythroid-2 related factor 2 (Nrf2), NAD(P)H dehydrogenase quinone 1 (NQO1), and superoxide dismutase-2 (SOD2), were significantly down-regulated in the kidney of *db/db* mice, while the oxidative marker levels, such as NADPH oxidases 2 and 4 (NOX2 and NOX4), were significantly increased, as shown in **Figure 7C**. However, interestingly, these changes were recovered to the normal level after the treatment of bFGF. These results were further validated at the protein level using western blot analysis, where we found that bFGF treatment corrected the decreased levels of Nrf2, NQO1, and SOD2 as well as the increased levels of NOX2 and NOX4 in the kidney of *db/db* mice (**Figures 7D, E**). Additionally, we also observed that *db/db* mice had significantly increased levels of pro-apoptotic proteins (caspase-3, cleaved caspase-3, and Bax) and decreased level of apoptotic protein (Bcl-2) in the kidney relative to age-matched wt mice (**Figure 7F**). Of note, the recovery of these protein marker levels was obtained after bFGF treatment. Furthermore, we quantified the ratios of cleaved caspase-3/caspase-3 and Bax/Bcl-2, which have been commonly used to assess cell apoptosis. The results showed that these two ratios were significantly increased in the kidney of *db/db* mice relative to age-matched wt mice (**Figures 7G, H**), suggesting an enhanced cell apoptosis. Yet, bFGF treatment can significantly reduce renal cell apoptosis in *db/db* mice. **Figure 8** illustrates that the protective effect of bFGF on diabetic nephropathy may be mediated by correcting metabolic disorders and suppressing oxidative stress in *db/db* mice.

## Discussion

Diabetic nephropathy is a common diabetic complication and seriously affect human health worldwide (Yang, 2019), but so far no effective treatment strategies can be available. In the present study, our results reveal that bFGF could be a promising drug for the treatment of diabetic nephropathy, as indicated by reductions in urinary albumin to creatinine ratio and renal fibrosis. To explore its potential therapeutic mechanisms, we performed an NMR-based metabolomics study to analyze metabolic changes in the kidney and urine of *db/db* mice after bFGF treatment. The results show that bFGF treatment altered metabolic phenotypes in the kidney and urine of *db/db* mice, mainly involving energy metabolism, methylamine metabolism, osmoregulation, and oxidative stress.

Kidney is an organ with abundant mitochondria and high energy demand, followed by heart in the body (Forbes, 2016). Therefore, diabetic nephropathy is usually accompanied by abnormal energy metabolism (Zhong et al., 2012; Wei et al., 2015; Tanaka et al., 2018). In the present study, relative to age-matched wt mice, we found a disordered energy metabolism in *db/db* mice, as indicated by significantly increased levels of tricarboxylic acid (TCA) cycle intermediates, such as citrate, fumarate, ATP, and AMP. Interestingly, abnormal energy metabolism can be recovered by bFGF treatment. Mitochondrial damage was considered as the hallmark of kidney injury (Galvan et al., 2017), and the TCA intermediates were identified as the potential biomarkers for impairment of mitochondrial function in the kidney of diabetic animals (Zhao et al., 2011; Jiang et al., 2019). We thereby hypothesize that bFGF has a protective effect on renal mitochondrial function. Creatinine, as an indicator of renal function, is formed exclusively from creatine in the body, and creatine is a nitrogen-containing organic acid that provides energy for maintaining normal cellular physiology (Akira et al., 2012). In this study, we found a significantly higher metabolic rate from creatine to creatinine in *db/db* mice compared with age-matched wt mice, but notably this abnormal metabolism can be recovered in *db/db* mice after bFGF treatment. Taken together, our results reveal that the protective effect of bFGF on diabetic nephropathy in *db/db* mice may be associated with energy metabolism regulation. In addition, amino acid metabolism is necessary to protein synthesis for cell growth and also supports for energy metabolism. In this study, we found a significant reduction of amino acid metabolism in the kidney of *db/db* mice relative to age-matched wt mice, as indicated by decreases in alanine, glycine, and glutamate. Yet, their levels were not restored after the treatment of bFGF, which may imply that the protective effect of bFGF on diabetic nephropathy could be not achieved through amino acid metabolism.

Methylamine metabolites are produced from the degradation of dietary choline to TMA, TMAO, and DMA by the gut microflora (Messenger et al., 2013). In this study, *db/db* mice had significantly higher DMA and TMAO levels than age-matched wt mice, whereas their levels were significantly decreased by bFGF treatment, indicating a recovery of methylamine metabolism. TMAO has been reported to be closely associated with decreasing renal function (Mueller et al., 2015; Tang et al., 2015; Missailidis et al., 2016). Tang et al. also found that chronic dietary TMAO exposure directly resulted in progressive renal fibrosis and dysfunction in animal models. Thus, our results suggest that methylamine metabolism may contribute to the reno-protective effect of bFGF in *db/db* mice.

Myo-inositol as an osmolyte plays an important role in normal cellular physiology (Carlomagno et al., 2012). Previous studies have reported that myo-inositol could be an indicator for tubular dysfunction and renal cell stress under hyperglycemia, and has a key role in the etiology of diabetes mellitus, particularly for diabetic nephropathy (Prabhu et al., 2005; Kanwar et al., 2008). Gil et al. reported that in the level of urinary myo-inositol excretion indicated severity of chronic kidney disease (Gil et al., 2018). In the current study, we found a significantly increased myo-inositol level in the kidney of *db/db* mice relative to age-matched wt mice; however, this abnormal increase can be significantly reduced after bFGF treatment. This finding suggests that the protective effect of bFGF on diabetic nephropathy in *db/db* mice may be implicated in myo-inositol-mediated mechanisms such as osmoregulation.

It is worth noting that taurine was identified to have the greatest contribution to bFGF-induced metabolic changes. Taurine is one of abundant free amino acids in mammals’ tissues and implicated in oxidative damage, endoplasmic reticulum stress, and apoptosis (Timbrell et al., 1995; Jong et al., 2017). Hence, taurine has been reported to protect against kidney injure (Adedara et al., 2017; Stacchiotti et al., 2018; Adedara et al., 2019). In the present study, *db/db* mice had a significantly lower level of renal taurine than age-matched wt mice, but its level was significantly increased after bFGF treatment. Our results also reveal that bFGF treatment corrected the decreased level of SOD, an antioxidant marker, and the increased level of MDA, a marker of lipid peroxidation, in the kidney of *db/db* mice. Additionally, significant reductions in apoptosis indicators were also detected in the kidney of *db/db* mice treated with bFGF. These findings suggest that the protective effect of bFGF on diabetic nephropathy may be also associated with taurine-mediated oxidative stress and apoptosis. Oxidative stress can increase ROS production and impair antioxidant capacity because of the generation of ROS exceeding the capacity of antioxidant defense system (Wilcox, 2005). Moreover, oxidative stress is believed to play an important role in cell apoptosis (Buttke and Sandstrom, 1994; Chandra et al., 2000) and also associated with diabetic complications (Ruiz et al., 2013; Yang et al., 2015). Nrf2 is a critical transcription factor to maintain redox homeostasis and regulates the expression of antioxidative stress genes including NQO1 and SODs (Dodson et al., 2019). It has been reported that hyperglycemia can induce oxidative stress and increase kidney injure *via* Nrf2 signaling (Wu et al., 2018). However, interestingly, our results show that bFGF treatment corrected the decreased levels of Nrf2, NQO1, and SOD2 in the kidney of *db/db* mice. In addition, NADPH oxidases (NOX2 and NOX4) are a family of enzymes that catalyze the generation of ROS, and have been linked with the development of kidney disease (Holterman et al., 2015). In this study, we found that the levels of NOX2 and NOX4 were significantly increased in the kidney of *db/db* mice relative to age-matched wt mice, but bFGF treatment recovered their levels to the normal level. Therefore, these findings reveal that bFGF may ameliorate oxidative stress and apoptosis during the development of diabetic nephropathy in *db/db* mice *via* NOX-ROS-Nrf2 signaling.

## Conclusion

In this study, bFGF treatment can effectively reduce urinary albumin to creatinine ratio and renal fibrosis as well as remodel metabolic phenotype in *db/db* mice. Metabolomics analysis identified that taurine, as an antioxidative metabolite, had the greatest contribution to bFGF-induced metabolic changes. Additionally, we found that bFGF treatment alleviated oxidative stress and apoptosis in the kidney of *db/db* mice though NOX-ROS-Nrf2 signaling. However, several limitations or further works should be considered: (1) The biological activities of bFGF are mediated by FGF receptors, so changes in bFGF and its receptors need to be further analyzed in the kidney; (2) A multi-analytical platform is recommended to detect more detailed metabolic pathway changes; (3) A multi-omics analysis will advance a better understanding of the therapeutic effect of bFGF on diabetic nephropathy and its potential mechanisms. Further translational studies are encouraged to modify bFGF structure for excluding its potent mitogenic and angiogenic activities, and facilitate its clinical application for the treatment of diabetic nephropathy in humans.

## Data Availability Statement

All datasets generated for this study are included in the article/supplementary material.

## Ethics Statement

The animal study was reviewed and approved by the Institutional Animal Care and Use Committee of Wenzhou Medical University.

## Author Contributions

HG and HZ contributed to experimental design. TW, JN, and SW contributed to animal experiments and metabolomics analysis. TW, QS, CL, and LZ contributed to molecular biology experiments and data analysis. HZ and TW contributed to result interpretation and writing. All authors have read, revised, and approved the final manuscript.

## Funding

This work was supported by the National Natural Science Foundation of China (nos. 81600653, 21605115, and 21974096).

## Conflict of Interest

The authors declare that the research was conducted in the absence of any commercial or financial relationships that could be construed as a potential conflict of interest.
